# Development and Testing of a Magnetically Actuated Capsule Endoscopy for Obesity Treatment

**DOI:** 10.1371/journal.pone.0148035

**Published:** 2016-01-27

**Authors:** Thanh Nho Do, Tian En Timothy Seah, Ho Khek Yu, Soo Jay Phee

**Affiliations:** 1 School of Mechanical and Aerospace Engineering, Nanyang Technological University, 50 Nanyang Avenue, Singapore, 639798, Singapore; 2 Department of Medicine, Yong Loo Lin School of Medicine, National University of Singapore and National University of Health System, Singapore, 119260, Singapore; National Cancer Center, JAPAN

## Abstract

Intra-gastric balloons (IGB) have become an efficient and less invasive method for obesity treatment. The use of traditional IGBs require complex insertion tools and flexible endoscopes to place and remove the balloon inside the patient’s stomach, which may cause discomfort and complications to the patient. This paper introduces a new ingestible weight-loss capsule with a magnetically remote-controlled inflatable and deflatable balloon. To inflate the balloon, biocompatible effervescent chemicals are used. As the source of the actuation is provided via external magnetic fields, the magnetic capsule size can be significantly reduced compared to current weight-loss capsules in the literature. In addition, there are no limitations on the power supply. To lose weight, the obese subject needs only to swallow the magnetic capsule with a glass of water. Once the magnetic capsule has reached the patient’s stomach, the balloon will be wirelessly inflated to occupy gastric space and give the feeling of satiety. The balloon can be wirelessly deflated at any time to allow the magnetic capsule to travel down the intestine and exit the body via normal peristalsis. The optimal ratio between the acid and base to provide the desired gas volume is experimentally evaluated and presented. A prototype capsule (9.6mm x 27mm) is developed and experimentally validated in *ex-vivo* experiments. The unique ease of delivery and expulsion of the proposed magnetic capsule is slated to make this development a good treatment option for people seeking to lose excess weight.

## Introduction

Obesity is a condition in which the body weight of a person is above a certain threshold, potentially leading to cardiovascular disease, musculoskeletal disorders, and even cancer. This happens when the calorific intake of food is higher than the energy expended by metabolism. According to a report from World Health Organization (WHO), the number of obese people worldwide has doubled since 1980. Due to modernization and change in lifestyle patterns, maintaining a balanced diet and regular exercises has become more challenging. Adults tend to consume high-calorie food in a larger quantity while leading a sedentary lifestyle, thus increasing chances of obesity. For individuals with more severe obesity, medical procedures such as the bariatric surgery and placement of intragastric balloon (IGB) are available. Bariatric surgery involves manual alteration of the stomach size or the digestive track by means of gastric bypass or by using gastric bands. These procedures are proven to be effective in promoting weight loss, but they are invasive and may cause adverse effects such as surgical complications, metabolic bone diseases, and kidney diseases [1].

A less invasive way for weight loss is to introduce an IGB which is filled with gas or liquid to occupy stomach space and hence reduce gastric volume and improve satiety [2]. The IGB is endoscopically placed inside the patient’s stomach through the mouth under conscious or unconscious sedation in an outpatient setting. Diagnostic endoscopy is performed to exclude any pre-existing upper gastrointestinal abnormalities. Under endoscopic vision, the IGB is inserted and filled with air or saline. Patients are required to remain under observation for while after the procedure. The removal of the IGB from body after the treatment period is also required to be performed under sedation with diagnostic endoscopy. A gastroscopic instrument such as a needle is used to puncture the IGB or suction through an extension tube applied to aspirate the contents of the IGB. The deflated balloon is grabbed using a grasping forceps or a polypectomy snare.

The first IGB to be commercialized was the Garren-Edwards Gastric Bubble (GEGB) [3]. Since then, a few IGBs have been developed and some of them are currently available in the market. Examples of currently available IGBs are Bioentrics Intragastric Balloon and Heliosphere [4, 5]. Although these products produced initially high efficacy in some clinical reports, the effectiveness for the weight loss decreased as a result of gastric adaptation over time [6]. In addition, commercial IGBs possess limitations on the insertion and the removal methods which can potentially result in patients’ discomforts and complications [7]. The IGBs have to be placed in the patients’ stomach using an endoscope or inflated using a tethering tube, causing abdominal discomfort, nausea, vomiting, and gastric mucous damage. Due to these limitations, the effectiveness of IGB as a generic weight-loss solution has not been widely accepted for now. Currently, the usage of traditional IGBs has been limited to a second choice modality for the group of patients who are not fit for surgery or the super obese who may require modest weight loss prior to a definitive intervention.

To overcome these limitations, a few developments of a wireless capsule robot for weight-loss treatment have been found in the literature. For example, Kencana et al. [8] developed an IGB in the form of an ingestible weight-loss capsule that would beswallowed with a glass of water. After the capsule has reached the patient’s stomach, a wireless signal activates an on-board electric actuator to inflate an attached outer balloon to a desired volume, which in turn occupies the gastric space. The action of stretching the gastric wall generates the feeling of satiety, thus limiting food intake of the patient. At the end of the treatment process, a wireless command is introduced to the capsule to deflate the balloon. However, the prototype could not be successfully miniaturised, having a diameter of 57mm and a length of 157mm. In a similar approach, Yan et al. [9] also proposed a smaller weight-loss capsule robot with a diameter of 30mm and a length of 80mm. A wirelessly controlled on board motor would mix chemicals together. Despite the advantages offered by on-board electronics in dynamically controlling the balloon size, limitations are introduced. On-board electronic mechanisms result in a larger capsule that is harder to swallow, or a reduction in chemical payload leading to a smaller, less effective balloon. Questions also remain about the safety of batteries and the power requirements of the on-board mechanisms. In addition, sizing guidelines based on the optimal chemical contents and clinical requirements are absent from the literature.

Magnetically actuated capsule robots have been widely studied in the literature [10–14]. In these approaches, small permanent magnets are placed inside a capsule robot and an external magnetic field is used to control the capsule locomotion with no on-board electric actuator and batteries, ample space can be reserved for the drug channels or biopsy mechanism. For example, Simi *et al*. [15] developed a miniaturized capsule mechanism for clipping and storing tissue from the gastrointestinal (GI) wall. A novel magnetic torsion spring rotates an inner cylindrical chamber of the capsule to distend the tissue into the chamber. The releasing magnetic spring reverts to its original closed position and clips off the protruding tissue. Kong *et al*. [16] also developed a similar mechanism that uses heated shape memory alloys to provide the torsional force. Sehyuk *et al*. [17] proposed an alternative approach for biopsy using deployable microgrippers. A flexible capsule is actuated by increasing an external magnetic field strength to open up a chamber filled with thermally sensitive microgrippers. Upon contact with the GI tract surface, they fold over like staples, capturing a small amount of tissue. These microgrippers are then collected by a wet adhesive patch on the capsule. Another capsule function that was studied was insufflation of the GI tract, which tends to collapse around a capsule endoscope. Toennies et al. [18] first used wireless insufflation of a tip mounted balloon to provide the CE camera with a clearer view of the GI tract. Later, Gorlewicz *et al*. [14] proposed a magnetically activated mechanism that would allow the controlled mixing of acid and base reactants from two separate chambers when insufflation is demanded. This relies on ball magnets that are initially attracted to and plug disc shaped ferritic ports, preventing the mixing of the reactants. When a strong external magnetic field is applied, the ball magnets are displaced and the two chambers are completely connected to form a closed channel. Despite the abovementioned studies on magnetic capsule endoscopes, none have been carried out for the express purpose of inducing weight loss through the inflation and deflation of an intra-gastric balloon.

In this paper, we introduce a new development of ingestible weight-loss capsule that contains no on-board electronics and electric actuators. Instead, internal magnetic mechanisms are actuated by external magnetic fields. As the source of actuation is externally provided, the size of the magnetic capsule can be kept smaller and more space is made for the chemical channels. Compared to current approaches in the literature [8, 9], there are no limitations on the power supply of the proposed weight-loss capsule. In addition, no complex control algorithms are required for the capsule operation and the capsule size could be smaller (our current prototype possesses a size of 9.6mmx27mm compared to Kencana *et al*. [8] with a size of 57mmx107mm and Yan et al. [9] with a size of 30mmx80mm). The use of magnetic actuation in the weight-loss capsule offers higher safety, lower cost, easier use, and provides more effective weight-loss treatment due to its dynamic administration process. In the long run, we envision having an over-the-counter magnetically actuated weight-loss capsule for obese and moderately obese people. Upon ingestion with water, the magnetic capsule will promote weight-loss by expanding and occupying the gastric space as an IGB, hence introducing the feeling of satiety. Oral administration eliminates hospitalization, recovery time, complications related to endoscopy or surgery, and mitigates long-term side effects from drugs. The design is also simple and low cost, making it a very viable commercial product. To validate the proposed approach, various experiments are carried out to determine the optimal ratio of chemical needed and the desired balloon volumes. Ex-vivo experiments on virtual stomach and biological environments (porcine stomach) are also performed to show the feasibility of the proposed magnetic weight-loss capsule.

## Materials and Methods

### Operating principle for the proposed MWCE

**Fig 1:**
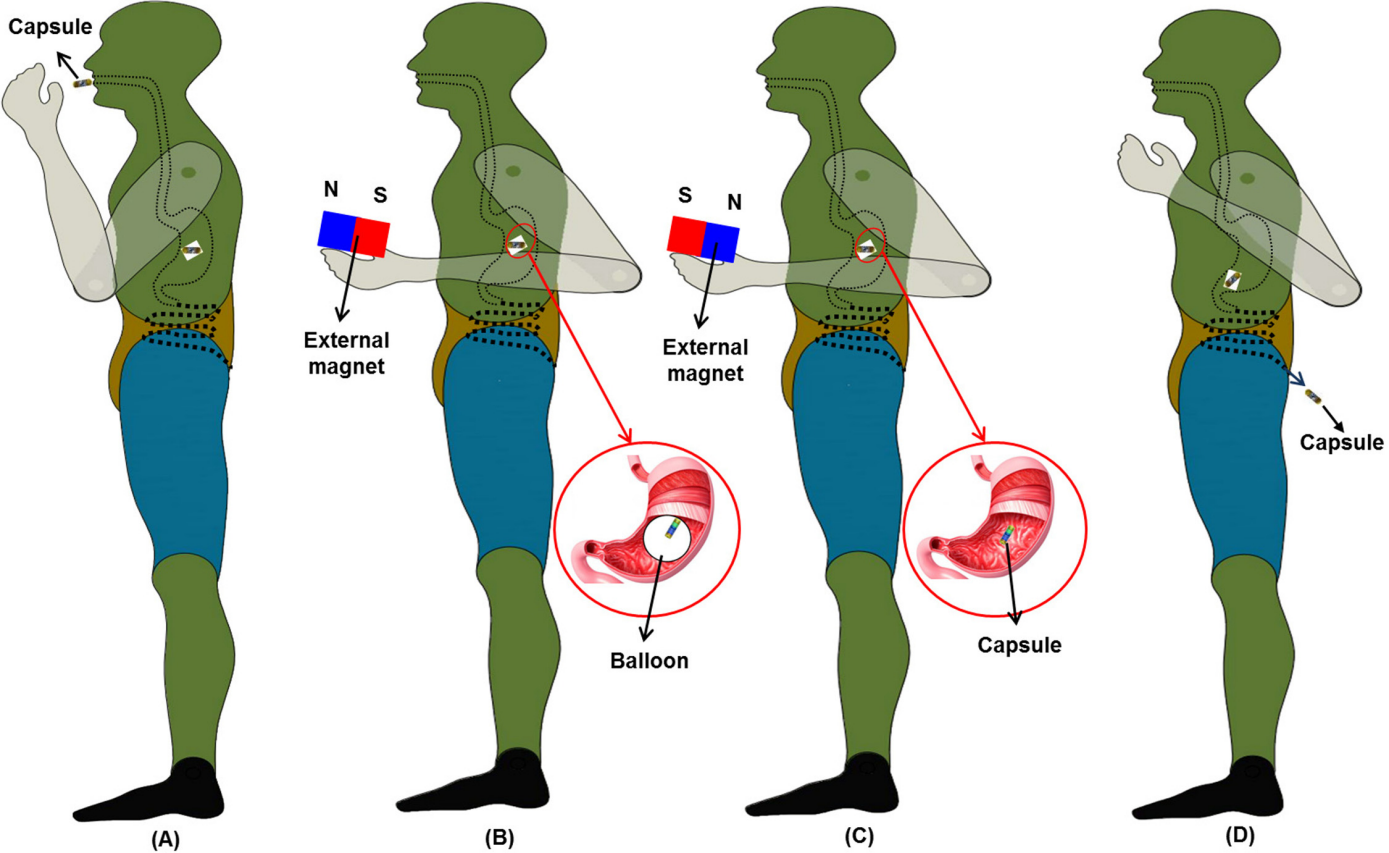
Four main stages for using the MWCE: (A) Swallow stage; (B) Inflation of the balloon; (C) Deflation of the balloon; (D) Excretion of the capsule out of the patient’s body.

The application scenario for the proposed MWCE is presented in Fig 1. There are four main stages for using the proposed MWCE: swallow, inflation, deflation, and excretion. The patient first swallows the capsule with a glass of water. After a period of around 6 seconds, the capsule reaches the patient’s stomach via oesophageal peristalsis [19]. The MWCE has two distinct acid and base chambers separated by an inflation valve. Once the capsule has reached the patient’s stomach (see Fig 1(A)), an external magnetic field generated by an external permanent magnet is introduced to open the inflation valve (Fig 1(B)). This external magnet can be mounted on a flexible structure that allows for manual operation. Inflation is achieved by having a chemical reaction between an acid and a base to generate the CO_2_ gas. The gas generated will thus inflate an outer elastic wrapping balloon to fill a certain space in the stomach and hence introduces the feeling of satiety.

After a predetermined period of treatment ranging from one to six months, the balloon is deflated using a secondary magnetically actuated mechanism similar to that of inflation. The external magnetic field triggers a deflation valve that causes the gas to exit the capsule into the stomach environment via the deflation valve (Fig 1(C)). The deflation mechanism can be differentiated by the direction of magnetic field applied. For example, a south pole of the external permanent magnet is used to trigger the inflation mechanism while its north pole is used for the deflation. Once the balloon is deflated, the capsule will be evacuated via stomach contractions and excreted naturally (Fig 1(D)). The detailed designs for the proposed inflation and deflation mechanisms will be introduced in the next sections.

### Biocompatible chemicals for balloon inflation

The use of an air-filled balloon has been proven to be a valid therapeutic option in the temporary treatment of obesity [20]. We now explore the potential of using biocompatible gas for the balloon inflation. A possible effervescent reaction for generating a gas is the decomposition of hydrogen peroxide (H_2_O_2_) into water (H_2_O) and oxygen gas (O_2_) [18]. Hydrogen peroxide is stable and requires the addition of a catalyst (synthetic or otherwise) to initiate the reaction. However, hydrogen peroxide is toxic at the high concentrations required to inflate the balloon thus it is not safe for use in the ingestible capsule [21]. Gas can also be generated by acid-base reactions in medical applications [14, 22]. Naturally occurring food additives such as citric acid (H_3_C_6_H_5_O_7_) or acetic acid (CH_3_COOH) can be used as an organic acid, and sodium bicarbonate (NaHCO_3_) or potassium bicarbonate (KHCO_3_) can be used as the base. With the exception of acetic acid which may cause skin irritation at high concentrations, the concentrations that can be used are not restricted by FDA good manufacturing recommendations [23]. Potassium bicarbonate does pose an additional health risk for people with renal failure as it may overload their potassium regulatory systems, thus causing cardiac problems [24–27]. However, these chemicals are contained inside the capsule and the balloon and therefore these risks are almost eliminated. In this paper, we decide to compare the four permutations of organic acid and base possible with citric acid (CA), acetic acid (AA), sodium bicarbonate (SB), and potassium bicarbonate (PB) to determine which is best suited for use in the proposed MWCE. The acid-base reaction produces carbon dioxide (CO_2_) gas, water, and an aqueous salt. For example, the stoichiometric reaction between citric acid (H_3_C_6_H_5_O_7_) and sodium bicarbonate (NaHCO_3_) produces trisodium citrate (Na_3_C_6_H_5_O_7_), carbon dioxide (CO_2_), and water (H_2_O). Water as a solvent is necessary to dissociate the ions and provide a medium for the reaction to occur, increasing the likelihood that the reaction will be completed. However, beyond a certain point, the marginal benefit from adding more water diminishes and it only occupies valuable space in the capsule. Thus, we also investigate the concentrations of acid that produce the most amount of carbon dioxide gas for the least amount of initial volume.

### Acid-base reaction experiments

The stoichiometric reaction ratios provide an indication of what quantities of reactants are needed to produce a certain volume of gas. However, the actual volume of gas produced is affected by various factors such as solubility of the reactants, the products, and the temperature. Therefore, we conduct a spread of experiments across the different acid-base combinations to determine the smallest volume of aqueous acid and solid base. The obtained result will be used to determine desired amount of gas and the optimal size of capsule chambers. Secondary considerations such as rate of reaction are also investigated.

**Fig 2:**
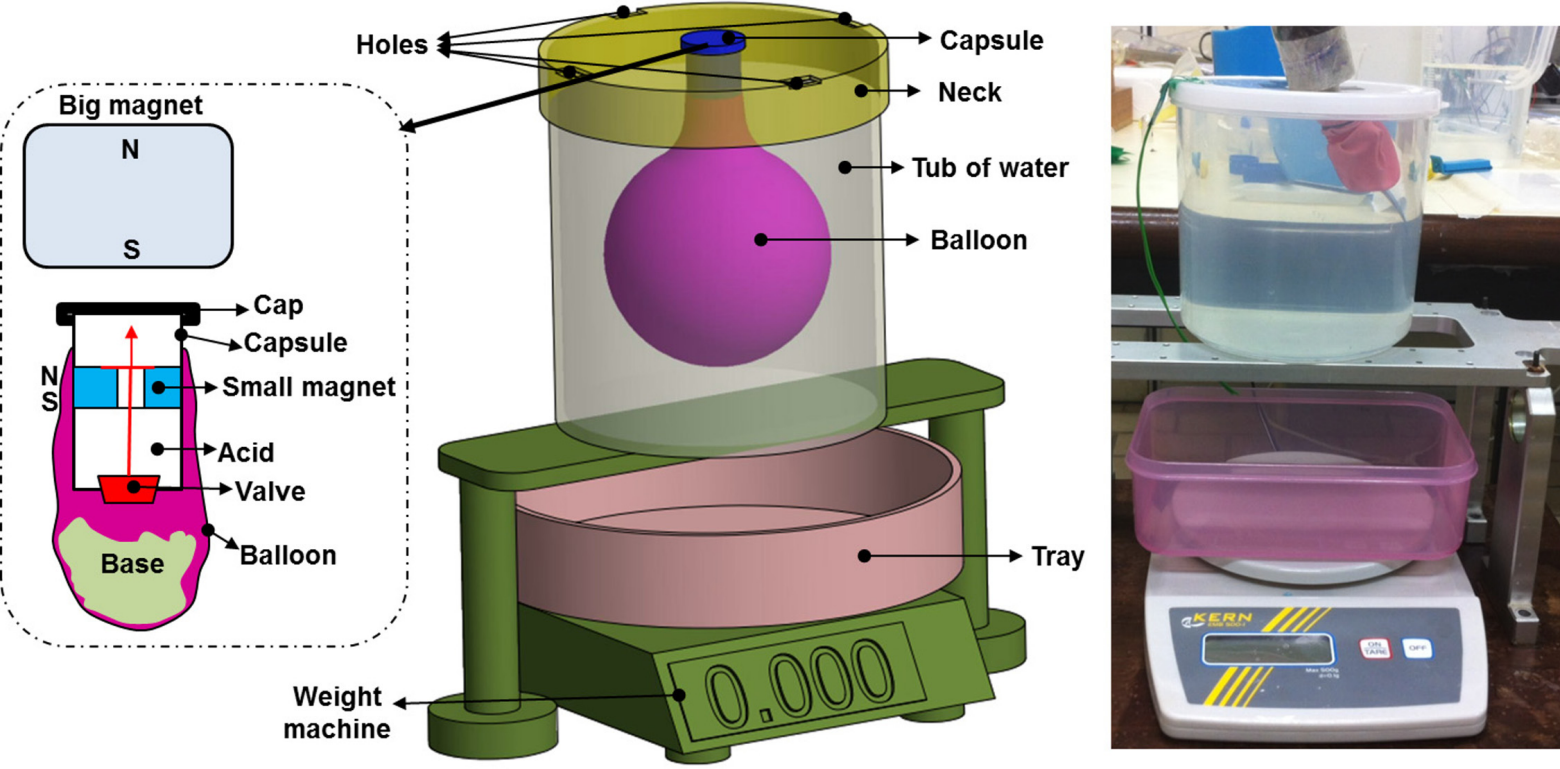
Acid-base experimental setup.

An experimental set up is established to characterize the acid-base reaction and volume measurement of the CO_2_ gas (see Fig 2). A deflated balloon was placed into a tub of water with the neck sticking out. Base powder was added to the balloon. A capsule with a magnetically actuated release valve was inserted into the neck of the balloon and secured with a cap. Upon actuation of the valve connected to a small inner magnet by an external big permanent magnet, the aqueous acid inside the capsule would flow down to the bottom of the balloon where it would react with the base powder. The expansion of the balloon due to the evolved CO2 gas would displace water from the tub via holes located at its neck, causing it to flow into a tray placed on a weight machine. Readings of the mass of water displaced were taken at 30 second intervals for the first 10 minutes, and the experiment was terminated after 15 minutes. The balloons were shaken periodically to ensure even mixing and to simulate patient motility after administration of the capsule.

### Volume multiplier

To assess the performance of the various acid-base and concentration combinations, the increase in volume of the balloon and initial volume of acid-base will be evaluated as parameters. The efficiency of acid-base reaction can be represented by:

$$\eta =\frac{V_{increase}}{V_{acid}+V_{base}}$$ (1)

where *V*_*increase*_ (ml) is the final volume of the balloon after the inflation, *V*_*acid*_ (ml) and *V*_*base*_(ml), are the volume of acid and base needed for the reaction, respectively.

For each acid-base combination, there exists an optimal amount of water solvent. For illustration, three different concentrations of acid for each combination were tested to investigate this effect. The acids and bases are from Sigma-Aldrich Corporation, USA. For an arbitrarily demanded volume of CO2 gas production, the stoichiometric masses and volumes of acid base combinations were calculated while distilled water was added to the acid to dilute it. The saturated concentration is observed around 59.2% for a powder citric acid [28]. Therefore, we used 60% as the maximum concentration during the experiments. For better illustration, solutions of 50% and 40% citric acid were also tested. In comparison, acetic acid exists as a liquid form which allows higher concentrations of 50%, 60%, and 70% to be used. This was done to make the initial volume of reactants comparable to that of citric acid. Table 1 shows the volumes of chemicals used in the experiments and the efficiency coefficient (*η*). It is assumed that the base powder in the capsule will be compressed to achieve as small a volume as possible. Note that five trials are carried out for each combination to ensure the repeatability and the presented results are mean values.

**Table 1:** Experimental results for different combinations of biocompatible acids and bases.

	CA			AA		
Concentration (%)	40	50	60	50	60	70
Volume of Acid (ml)	0.845	0.660	0.542	0.831	0.696	0.600
Total Volume (ml) of Acid and 0.2163 ml of PB	1.06	0.874	0.756	1.05	0.910	0.813
*η*	100.328	114.181	118.035	82.865	90.122	88.527
Total volume (ml) of Acid and 0.2579 ml of SB	1.10	0.918	0.800	1.09	0.954	0.858
*η*	100.923	105.841	110.25	70.407	67.493	59.001

### Chemical reaction results

**Fig 3:**
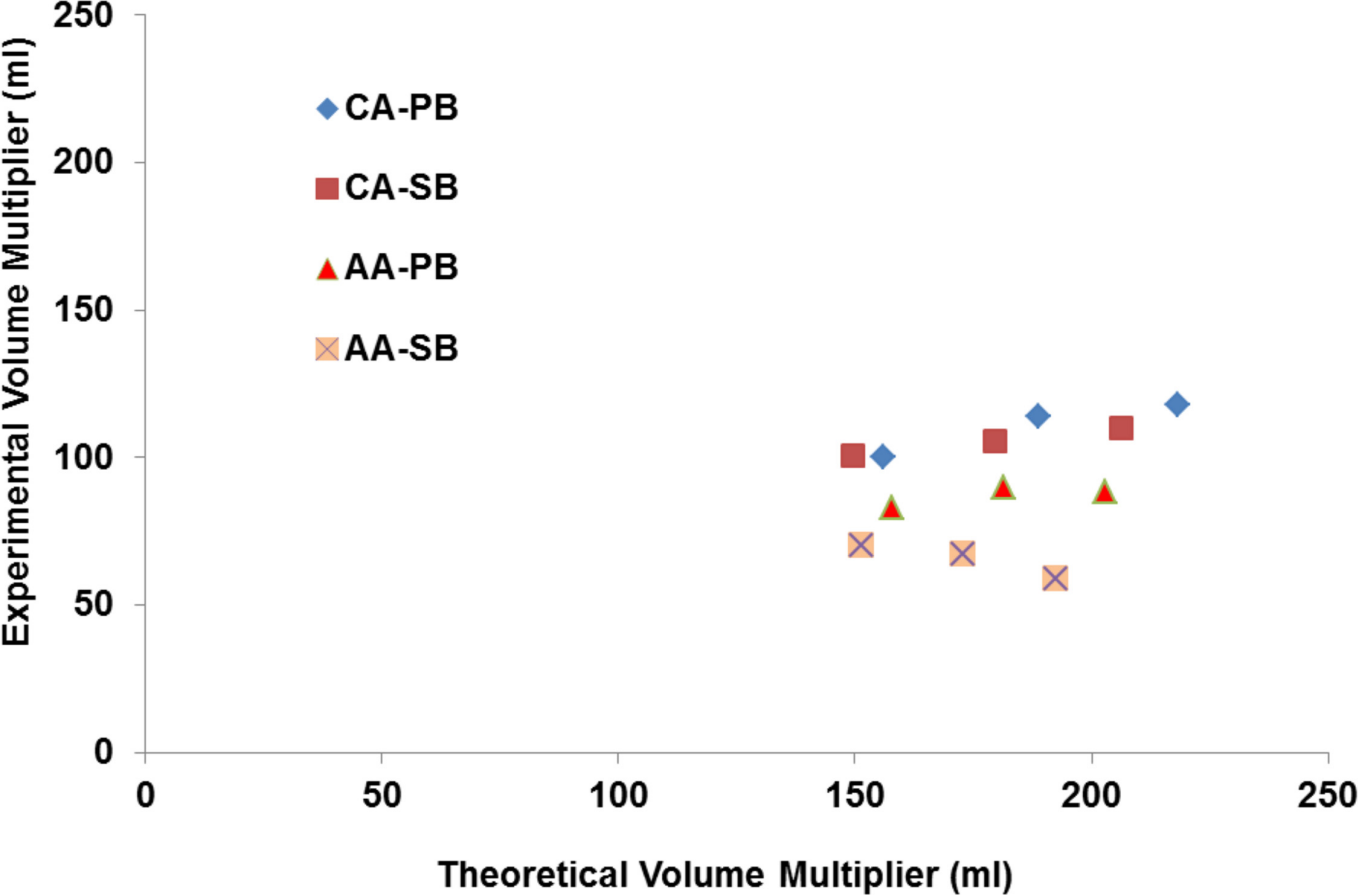
Measured volume multiplier after 15 minutes compared with the expected stoichiometric values. The points in the CA series, from left to right, represent 40%, 50% and 60% acid concentrations, respectively. The points in the AA series, from left to right, represent 50%, 60% and 70% acid concentrations, respectively.

Fig 3 presents the comparison results between the real-time experiments and theoretical calculation for different combinations of acid and base. It is noted that the real-time measurements are carried out within 15 minutes of reaction. We also observed that after 15 minutes of the reaction, further increases in the measured volumes are not significant for most of combinations. The results given in Fig 3 and Table 1 verify that the citric acid combinations are shown to be superior and the best performance is observed for 60% citric acid and potassium bicarbonate (highest value of *η* = 118.035). The acetic acid and sodium bicarbonate combination fared the worst. Hence, 60% of CA and PB are chosen to be the chemical combination for use in the capsule. It is also noted that the low water content causes an incomplete reaction, but it is still the most efficient combination. Other possible reasons for deviation from the theoretical volume are the unreacted base powder adhering to the sides of the balloon or CO2 gas remaining dissolved in the solution due to the pressure of the balloon.

**Fig 4:**
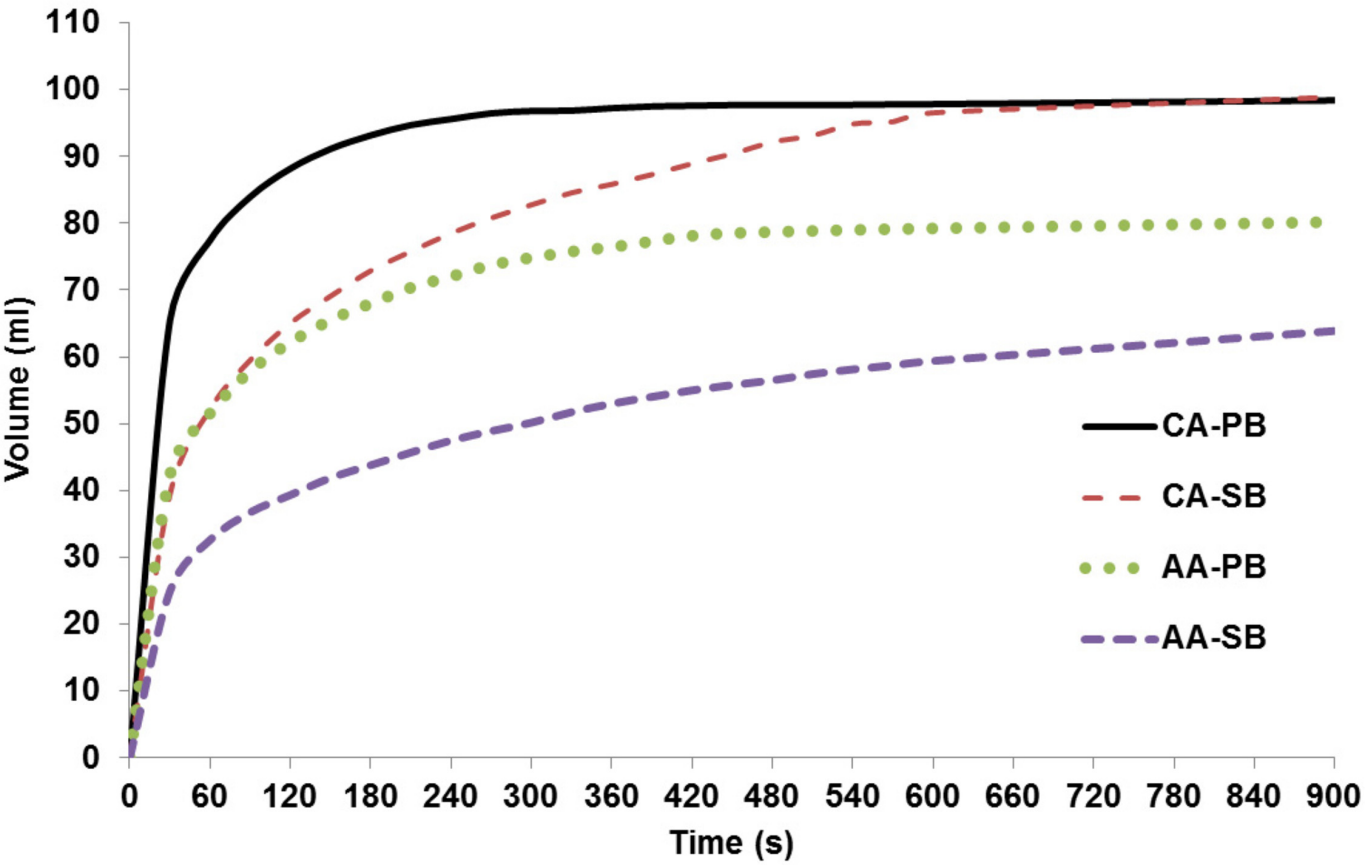
Carbon dioxide produced over time for each type of acid-base combinations.

The reaction rate results which are shown in Fig 4 are also evaluated in the experiment. It can be seen that CA-PB reaches equilibrium the fastest, and that the reaction of AA-SB is the slowest, which partially explains its low volume multiplier at the mark of 15 minutes. However, since CA-PB and CA-SB have comparable equilibrium volumes, it can be inferred that AA-PB and AA-SB will end up with similar final volumes given enough time. As the balloon needs to reach a minimum size after 15 minutes to prevent evacuation from the stomach, it is advantageous to choose the fastest reaction since real-world conditions may not allow for optimal mixing in the capsule. For example, the use of compacted powder may slow down the reaction. These findings further support the use of CA-PB in the proposed capsule.

### System design for the proposed magnetic capsule

#### Detailed structure of the proposed capsule

**Fig 5:**
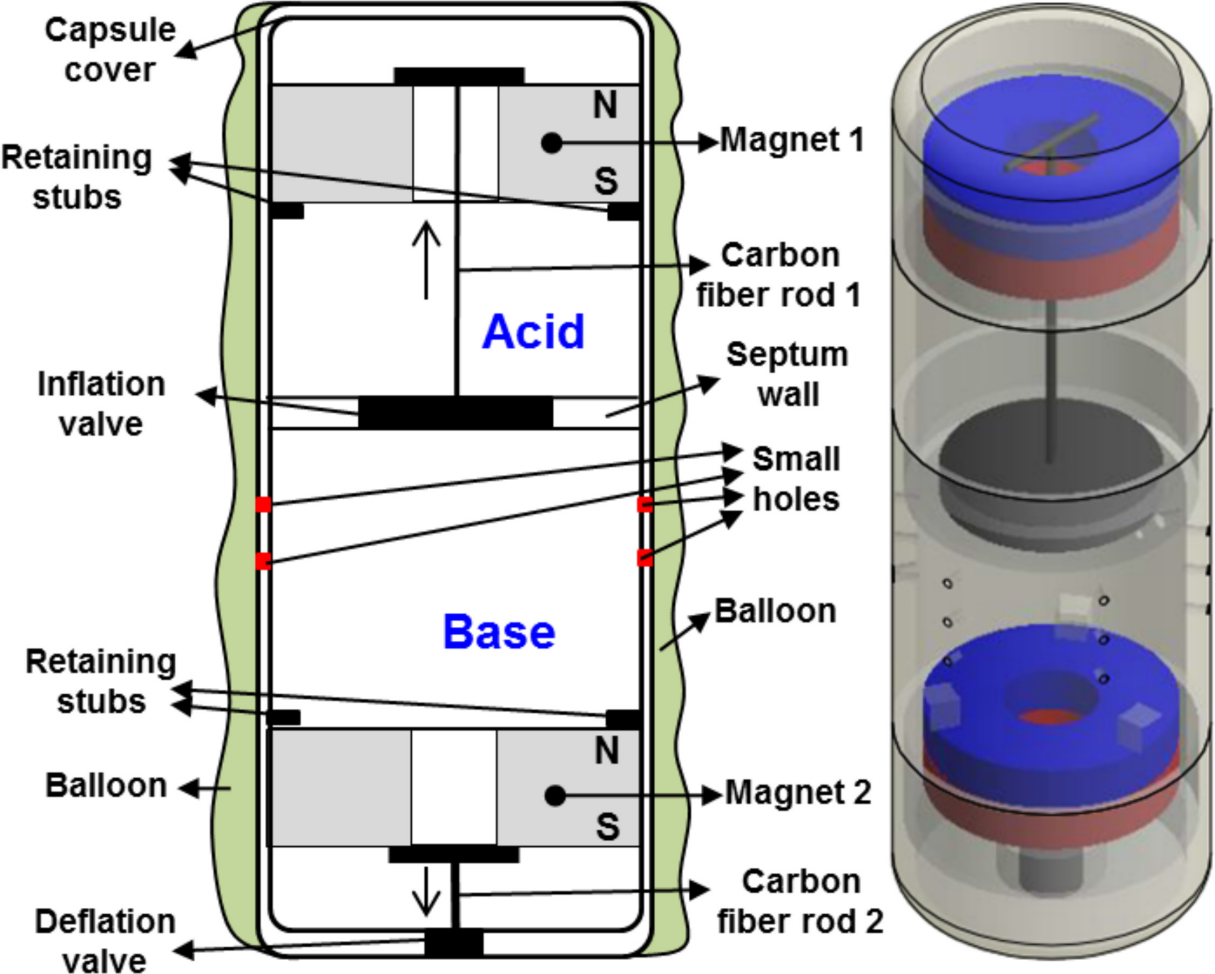
Structure of the proposed MWCE.

The proposed MWCE which is shown in Fig 5 includes an inner capsule, a balloon wrapped around the outside of the inner capsule, a liquid acid stored inside the one of the inner chambers of the capsule, a powdered solid base stored inside the other chamber of capsule, two small permanent magnets 1 and 2, inflation mechanism, and deflation mechanism. The inner capsule contains two separated chambers isolated by the inflation valve that has a compliant silicone element and a rigid carbon fiber rod 1. The acid chamber contains aqueous solution of acid and it is made out of an inert polypropylene-like material. All mechanisms in contact with the liquid have been treated to be similarly inert. The silicone element is connected to a ring neodymium magnet 1 by a rigid carbon fiber rod 1. The magnet may be Teflon coated for chemical inertness and ease of sliding inside the chamber. It is orientated with the North Pole facing the top of the capsule. Sufficient space exists between the magnet and the distal end of the chamber to allow the sliding of the magnet 1 and its connected components (carbon fiber rod 1 and inflation valve) along the longitudinal axis of the capsule. The magnet 1 only moves when a suitable level of attraction force from external magnet is applied to the capsule. Retaining stubs are also moulded into the inner surface of the acid chamber to limit the axial extent of motion towards the magnet 2. Initially, the magnet 1 rests against the retaining stubs and the silicone element forms part of the septum, creating a watertight seal.

The base chamber contains solution of a powder base chemical for reaction with the acid. It has small ventilation holes around the side to allow the CO_2_ gas produced by the acid-base reaction to inflate the outer balloon. The powder has been compacted to reduce its volume and to minimise its migration out of the ventilation holes. The base chamber also contains the deflation mechanism, which consists of another ring neodymium magnet 2 oriented in the same direction as the magnet 1. As with the inflation magnet 1, retaining stubs constrain its initial position. A small attractive force will exist between the two magnets at a distance *L*_*m1m2*_, holding them in place during transport and ingestion of the capsule, thus preventing accidental activation of the mechanisms. There is an air gap between the deflation magnet and the bottom of the base chamber, which allows the motion of the magnet 2 downwards when a suitable level of attraction force from external magnet is applied to the capsule. The bottom of the base chamber contains a hole that is stoppered by a silicone inflation valve. This valve will be opened if the magnet 2 moves towards the distal tip of the capsule.

The outer balloon is made out of an elastic biocompatible material such as silicone or rubber. In its initial state, it is folded or collapsed against the outside of the inner capsule. It is sealed to the inner capsule near the distal tip of the base chamber. Upon the chemical evolution of CO_2_ gas due to the acid-base reaction, it will expand into a desired volume. The balloon is expected to retain a desired volume up to 6 months, after which it will be magnetically deflated by the deflation mechanism.

#### Detailed operation principle for the inflation process

**Fig 6:**
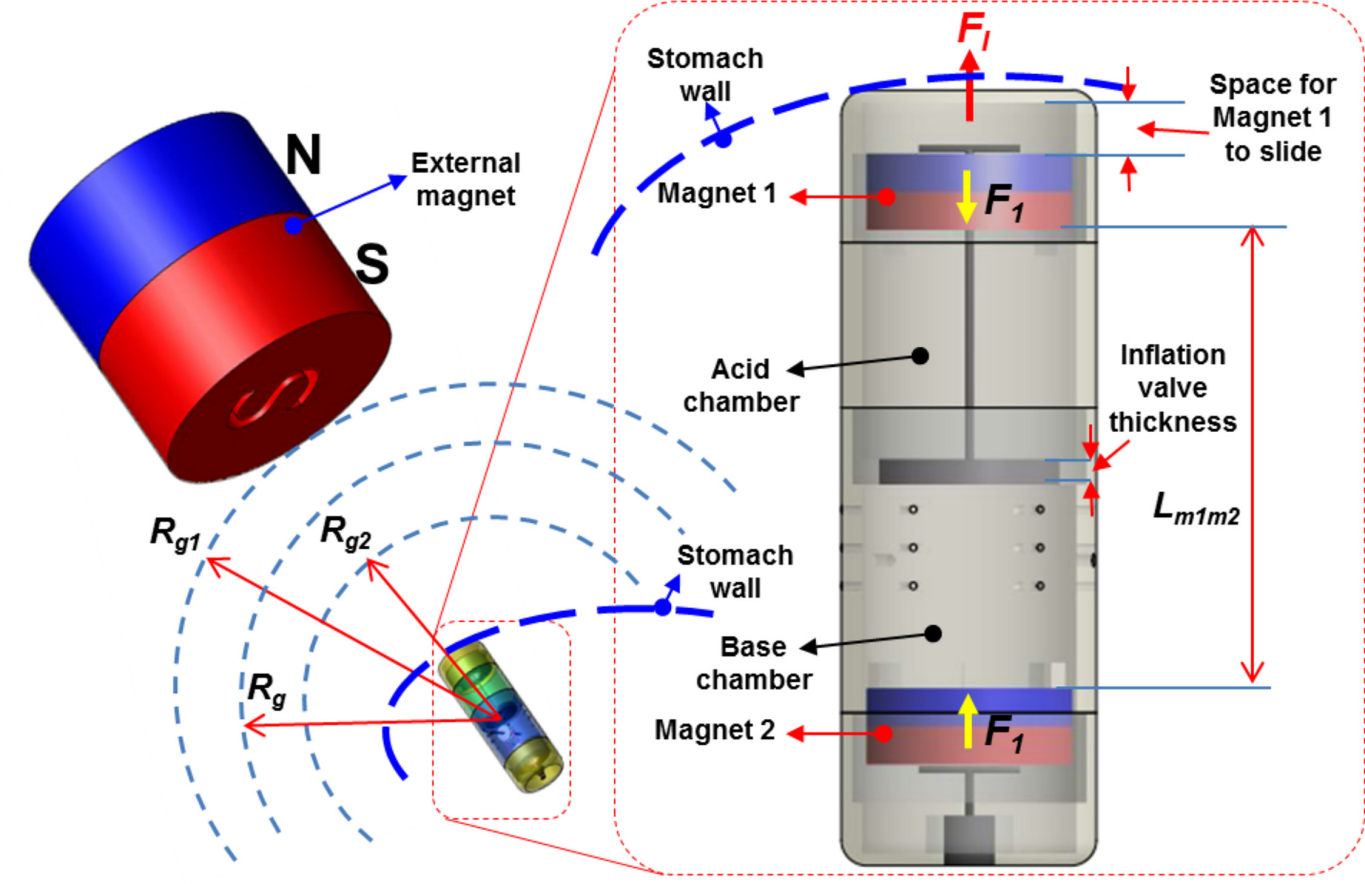
Operation principle for the inflation process.

To inflate the outer balloon, the inflation valve must be opened to mix the acid and the base together. Suppose that the South Pole of the external magnet represents the inflation process while its North Pole is used for the deflation process. The working principle for the inflation stage is shown in Fig 6. The external magnet which is manually manipulated by the patient is gradually brought closer to the stomach, leading with its South Pole. Let *R*_*g*_ be the distance measured from the free-floating capsule to the external magnet (Fig 6). When *R*_*g*_ falls below *R*_*g1*_, the magnetic force exerted on the capsule will cause it to move towards and automatically align itself in the same North-South orientation as the external magnet. This will cause its distal inflation tip to be braced against the inner lining of the stomach wall. If *R*_*g*_ is less than *R*_*g2*_, the force on the inflation magnet will overcome the frictional force between the inflation valve and the rest of the septum wall. If *R*_*g*_ has not yet decreased below R_g2_, the external magnet continues to approach the stomach wall anyway, until *R*_*g*_<*R*_*g2*_. Then the magnetic force causes distal magnet 1 and the inflation valve to move axially, breaking the seal and releasing the acid from the liquid containing chamber into the powder containing base chamber. At this phase, the attraction force to the magnet 1 reaches *F*_*I*_. As a result, the CO_2_ gas generated by the reaction will inflate the balloon to a desired volume via small holes in the base chamber. The detailed calculation for the attraction force *F*_*I*_ will be given in next sections.

#### Detailed operation principle for the deflation process

**Fig 7:**
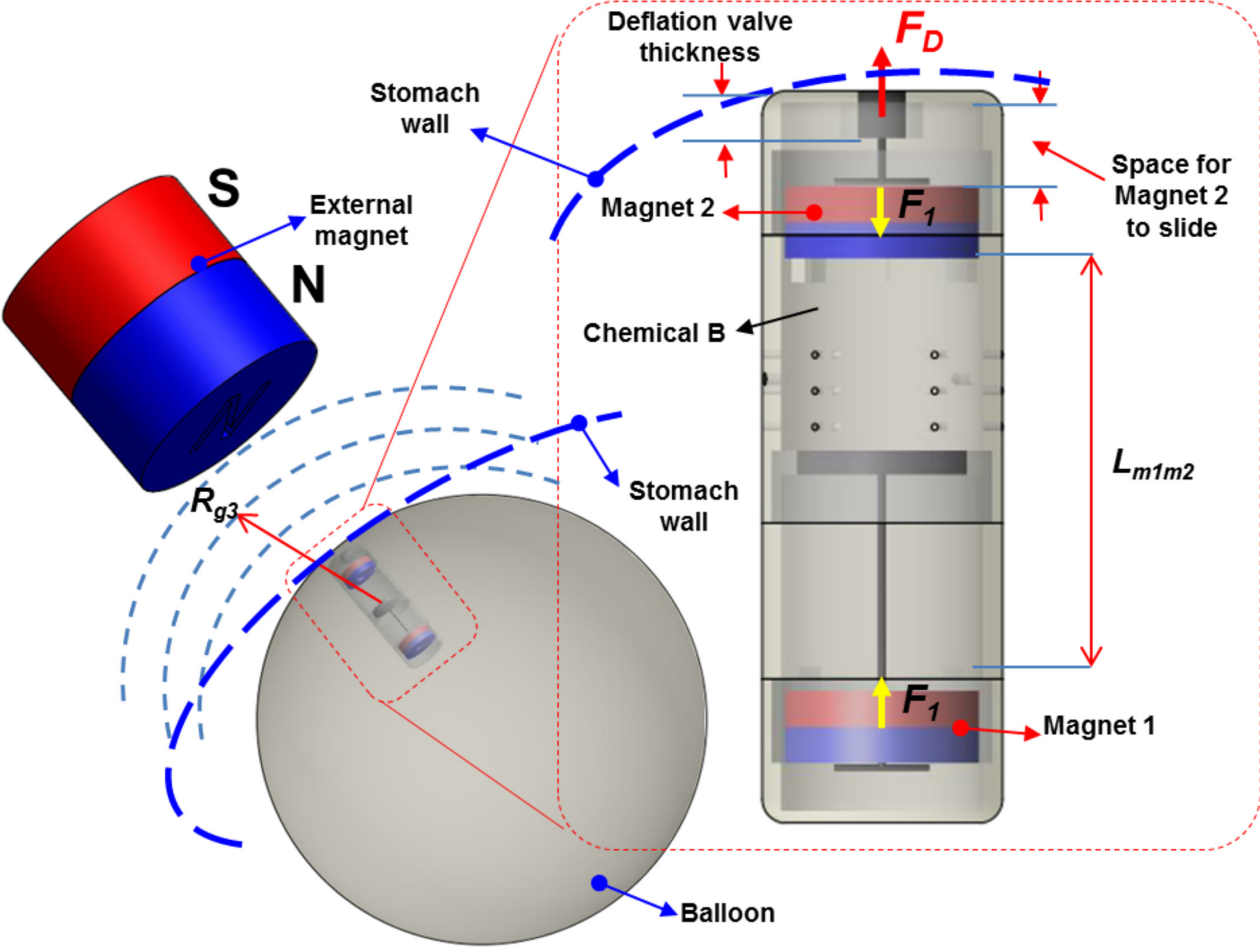
Operation principle for the deflation process.

To deflate the balloon after a period of treatment, the North Pole of the external magnet will be pointed and brought closer towards the patient’s stomach. The working principle for the inflation stage is shown in Fig 7. The capsule will automatically orientate itself to align with the deflation mechanism pointing towards the external magnet. The external magnet is then brought closer to a distance less than *R*_*g3*_, at which point the deflation magnet 2 moves and displaces the deflation valve via the carbon fiber rod 2, allowing air to leak out of the balloon to the stomach environment. At this phase, the force applied to the magnet 2 reaches *F*_*D*_. The deflated balloon and the capsule then travel through the pyloric sphincter and the rest of the alimentary canal before being passed. The detailed calculation for the attraction force *F*_*D*_ will be given in next sections.

## Results

### Magnetic weight-loss capsule prototype

**Fig 8:**
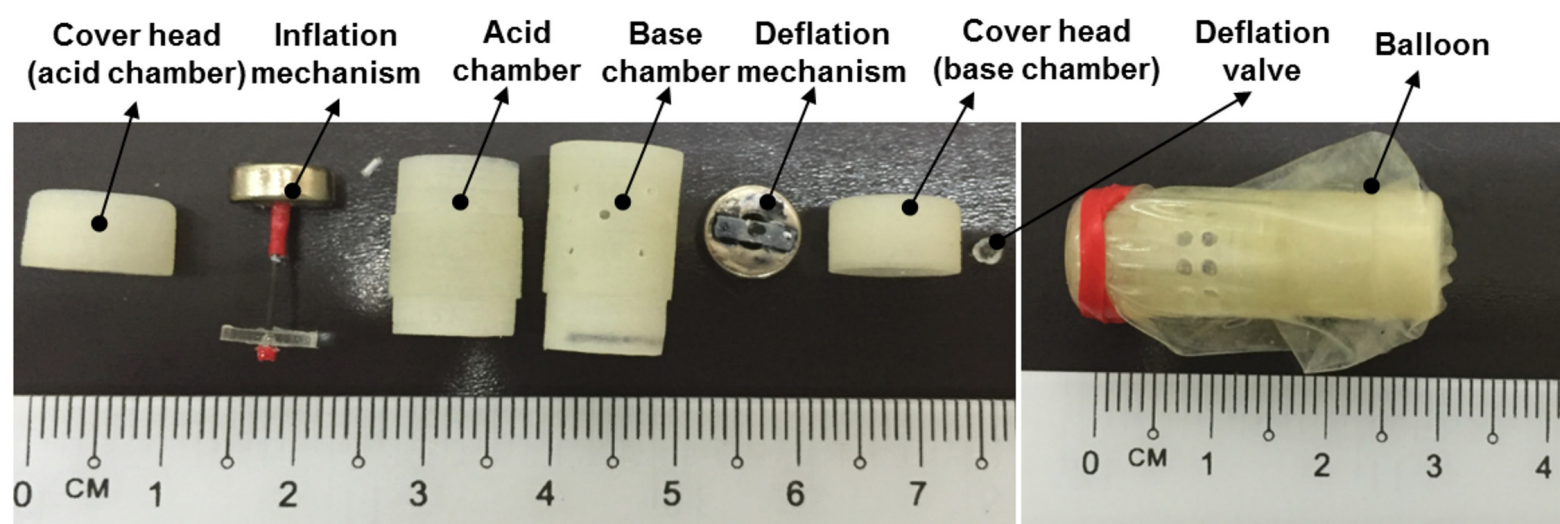
Photos of the MWCE and its outer balloon.

Using the obtained results from the aforementioned experiments, a prototype of the MWCE is developed. To keep the balloon capsule inside the human stomach during the treatment period, a minimal size of the inflation balloon is needed to prevent it from passing through the pyloric sphincter. It was shown that the normal diameter of pyloric sphincter in normal human is around 12.8±7mm and 16±8mm for gastric ulcer patient [29–31]. Assume that the inflated balloon has a sphere shape with its radius *D*_*balloon*_. For safety, our minimum requirement for the inflation balloon diameter is at least 40mm. A total volume of 1.084ml of acid (CA 60%) and 0.4326ml of base (PB) (see Table 1) will produce a volume of *V*_*increase*_ = 178.5ml of CO_2_ gas. With this obtained volume, the balloon diameter *D*_*balloon*_ = $2\sqrt[{3}]{3V_{increase}/(4\pi )}$ = 69.857mm. It can be seen that the balloon is guaranteed to stay inside the human stomach without passing through the pyloric sphincter. Fig 8 shows the photograph of a complete capsule prototype having an approximate outer diameter/length of 9.6m/27mm and its outer balloon. The whole capsule and its components are made by powder 3D-printer SLM®500HL from SLM Solutions Group AG, Germany. In this paper, a latex balloon is used as a representative of the IGB. The detailed specification for the proposed capsule prototype is presented in Table 2.

**Table 2:** Specification of the MWCE prototype.

***MWCE enclosure:***	
Outer/Inner diameter	~9.6mm/9mm
Length	~27mm
Inflation thickness valve	~1mm
Acid chamber volume including small magnet and 0.3mm of wall thickness/its length	~1143mm^3^/~18.5mm
Base chamber volume including small magnet and 0.3mm of wall thickness /its length	~491.5mm^3^/~8mm
***Internal ring magnets:***	
Shape/Material	Ring/NdFeB (N52)
Outer/Inner diameter/Thickness	8mm / 3mm / 3mm
***Other parts:***	
Diameter of PDMS Inflation valve/its thickness	~7mm/1mm
Diameter of PDMS Deflation valve/its thickness	~1.5mm/2mm
Diameter of carbon fiber rod	~0.5mm

### Force calculation for the proposed magnetic mechanisms

**Fig 9:**
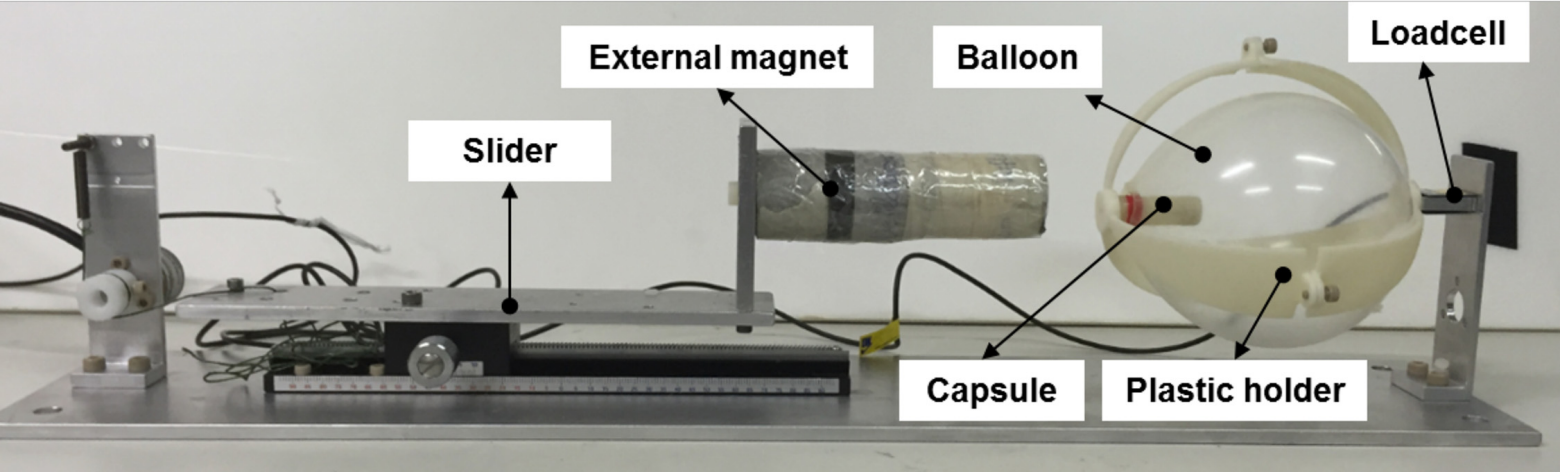
Experiment setup for force measurement.

An experimental setup is developed to determine the minimum force needed for opening the inflation and deflation valves (see Fig 9). The proposed MWCE is filled with 1.084ml of CA (60%) and 0.4326ml of PB in the acid chamber and the base chamber, respectively. An external magnetic field from a permanent magnet (NdFeB/N52) is used to open the inflation valve and produce the CO_2_ gas after the reaction. The permanent magnet is a cylinder-type with a diameter of 20cmm and a length of 70mm. The proposed capsule is connected to a FUTEK loadcell LSB200 to provide the force information. The detailed description for selecting the external magnet will be given in next sections. To hold the capsule and its inflated balloon after the reaction, a special plastic holder which is made by 3D-printer Fortus 250mc from Stratasys is used (see Fig 9). The external magnet is mounted on a slider that allows its forward and backward motion with respect to the loadcell position. The recorded signal is decoded using NI cRIO-9076 and LabVIEW software from National Instruments. After 15 minutes of the reaction, the obtained volume of the capsule balloon is measured using the experimental setup given in previous sections. To ensure the repeatability, five trials are carried out for the measurement of the inflation force, deflation force, and inflated balloon volume (see Table 3). It can be seen that mean forces of around 0.368N and 0.392N are needed to open the inflation and deflation valve, respectively. These forces will be used to estimate the external magnet size. Using the proposed experimental set up, we also measured the interaction force between the two internal magnets at a distance of *L*_*m1m2*_ = 18mm. The obtained force measurement *F*_*1*_ is approximate 0.022N. The interaction force between the two internal magnets is quite small compared to the interaction force between the internal small magnet and external magnet. Therefore, this small force can be ignored in the calculation of external magnet size. Note that the measured volume of the balloon is smaller than that in previous experimental validations for the first three trials. We observed that the base was trapped inside the base chamber and a small amount of the acid was push out of the capsule. In the last two trials, a modification on the capsule design was carried out and the balloon volume was increased. This problem will be discussed in the discussion section.

**Table 3:** Force and balloon volume measurement for the proposed capsule.

Trials	Inflation force *F*_*I*_ (N)	Deflation force *F*_*D*_ (N)	Obtained volume (ml)
1	0.4	0.32	141.2
2	0.31	0.45	143.5
3	0.45	0.39	146.8
4	0.29	0.37	169.3
5	0.39	0.43	170.1
Mean	0.368	0.392	154.18

### Estimation of the external magnet size

The size of the external magnet required to activate the inflation and deflation mechanisms is a function of the force required to release the elastomer valve and its distance from the capsule. The force required has been shown experimentally to be 0.368N to 0.392N. A numerical simulation was conducted using COMSOL Multiphysics 5.0 to determine the optimal shape and size of the external magnet. The strongest grade of neodymium, N52, was used in both the capsule and the external magnet. COMSOL applies the Amperian loop model where ∇ ∙ *B* = 0 to each finite node. This means that the magnetic field lines entering and leaving a node are equal [32]. It is noted in the simulation that an internal magnet is used for the calculation instead of two magnets. The use of single internal magnet does not affect the result since the distance between the two small magnets in the capsule is fixed and their interaction force is quite small. Only the force applied to the stomach wall is varied. However, our main concern is on the interaction force between the inner magnet and the external magnet. For the neodymium regions, the magnetic flux density *B* is given as:

$$B=\mu_{0}\mu_{r}H+B_{r}$$ (2)

where *μ*_0_ = 1 is the permeability of free space and *μ*_r_ = 1.05 is the relative permeability of neodymium. *B*_*r*_ = 1.42(T) is the remanence of a fully saturated N52 permanent magnet. *H* is the magnetic field strength (A/m).

The force *F* exerted on the small magnet is calculated using the Maxwell stress tensor. The forces acting on the surface elements of the small magnet are summed up:

$$F=\frac{1}{\mu_{0}}\oint_{A}\left(B\left(Bn\right)-\frac{1}{2}B^{2}n\right)\;dA$$ (3)

where *n* is a vector normal to the surface area element.

A spheroid magnet of radius 150 mm (volume 14100 *mm*^3^) was defined as the control, as it would have the maximum theoretical surface field strength. However, for the capsule, a cylindrical external magnet is preferred due to better uniformity of the magnetic field in the axial and radial directions. This means that the inflation mechanism can be triggered even if the precise location of the capsule is unknown. The flat surfaces also allow the external magnet to be pressed closer against the abdomen, which should compensate for the weaker axial magnetic field compared with a sphere. These effects were quantified by simulating cylinders of volume 14100 mm^3^ but with different aspect ratios. Two types (cylinder and sphere) of external permanent magnets are used for the calculation (see Table 4). The internal magnet is a cylinder type with thickness of 3mm and diameter of 8mm. To simplify the simulation process, the ring magnet is substituted by a cylinder magnet. This substitute does not significantly affect the result because the simulation is only used for the estimation of external magnet size. Let *L*_*ext*_ be the length of cylinder magnet and *R*_*ext*_ be the radius of the magnet (both cylinder and sphere), different air gaps of 150mm, 100mm, 50mm and 20 mm between the small magnet and external magnet were used to evaluate the designed external magnet. It is also noted that forces *F*_*150*_, *F*_*100*_, *F*_*50*_, and *F*_*20*_ represent the forces at the air gaps of 150mm, 100mm, 50mm, and 20mm, respectively.

**Table 4:** Simulation results for different shapes, sizes, and distances of the external magnet.

Shape	*L*_*ext*_ (mm)	*R*_*ext*_ (mm)	*F*_*150*_ (N)	*F*_*100*_ (N)	*F*_*50*_ (N)	*F*_*20*_ (N)
Sphere	-	150	0.16	0.38	0.92	1.70
Cylinder	150	173.2	0.19	0.31	0.39	0.40
Cylinder	200	150	0.22	0.35	0.54	0.59
Cylinder	300	122.5	0.21	0.39	0.67	0.82
Cylinder	400	106.1	0.19	0.36	0.74	1.00

**Fig 10:**
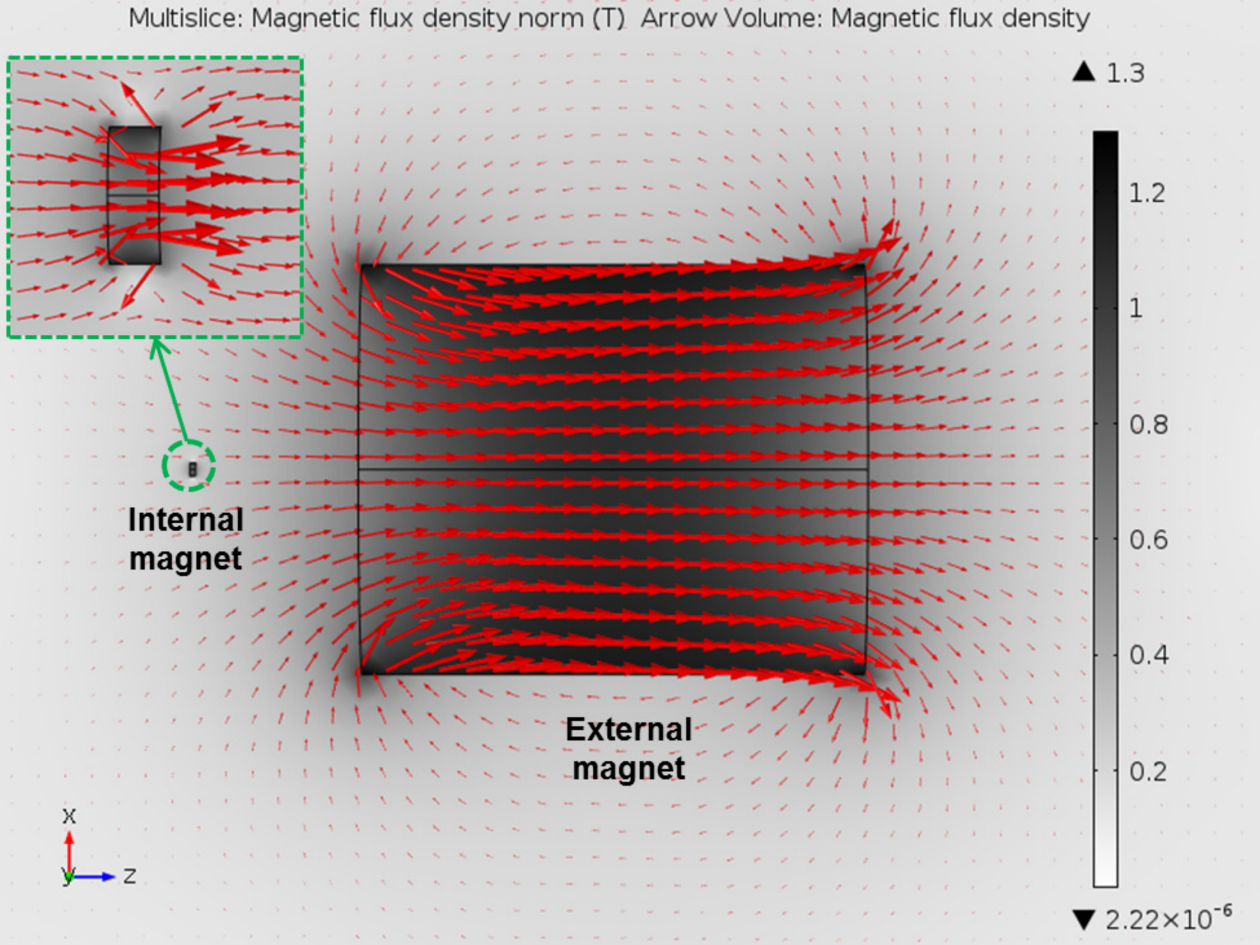
Simulation results for the estimation of external magnet size using COMSOL Multiphysics 5.0.

The results from Table 4 show that while a sphere does indeed exert the greatest axial force at close range, it exhibits a drastic drop off in force with increasing distance. It has been experimentally measured that the inflation force has been experimentally measured to be around 0.368N–0.392 N. If the external magnet is expected to operate at a distance of 100 mm from the capsule, a sphere magnet with a minimum diameter of 300 mm or a cylinder magnet with minimum length of 300 mm and diameter of 245mm will exert enough force. As discussed in previous parts, the 300 mm long cylinder is chosen to ensure patient safety by reducing the potential peak force exerted on the capsule. Even if the external magnet was to approach the capsule to a distance of 20 mm, the total force would be 1.64 N (summing up forces due to both magnets inside the capsule). The simulation result for a cylinder magnet with length of 300mm and diameter of 245mm is shown in Fig 10.

### Real-time validation results

**Fig 11:**
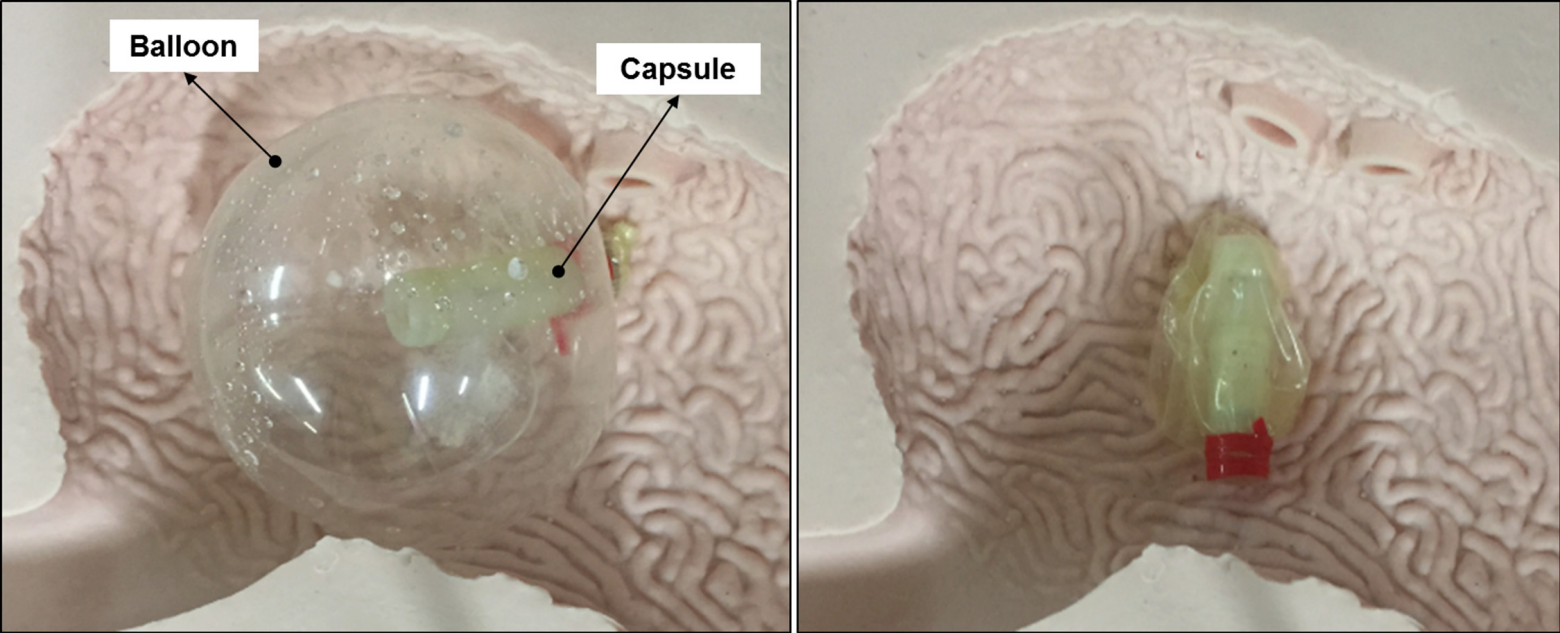
Validation results for the proposed MWCE on a virtual stomach EMS from The Chamberlain Group, USA. The left figure shows the balloon is inflated and the right shows the balloon is deflated to a compact size.

**Fig 12:**
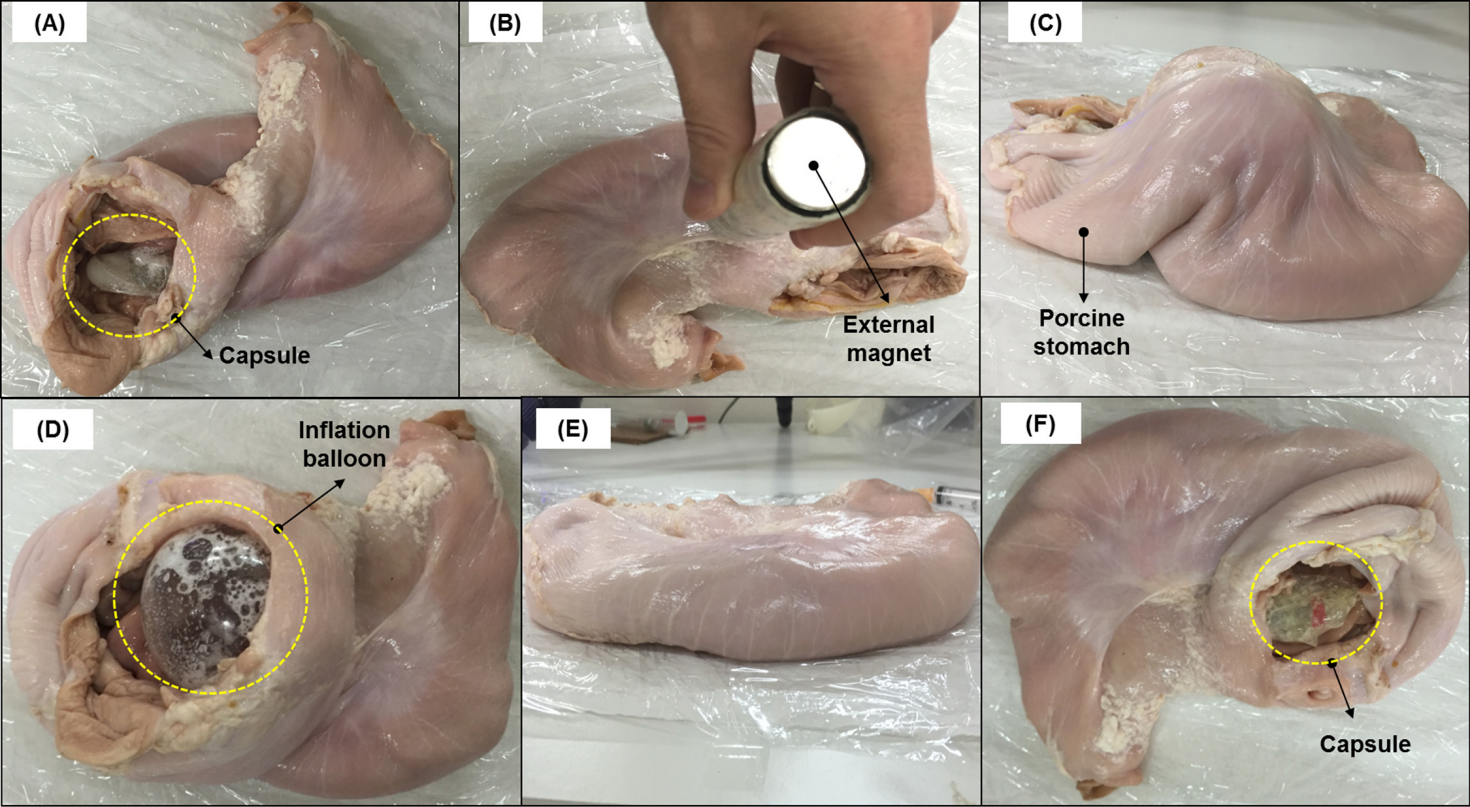
Validation results for the proposed MWCE on a porcine stomach. (A) Inserting the capsule-balloon into a porcine stomach. (B) The external magnet approaches the porcine stomach to inflate the balloon. (C) The balloon is inflated inside the porcine stomach after 15 minutes of the reaction. (D) The inflation balloon is partially exposed on the porcine stomach. (E) The balloon is deflated to its original compact size. (F) The deflation balloon and its inner capsule are partially exposed on the porcine stomach.

Two sets of experimental validations are conducted on the proposed MWCE. The first experiment is carried out on a virtual stomach EMS from The Chamberlain Group, USA. Although it is known in theory that most biological tissues possess similar magnetic permeability to the air, a similar biological environment should be carried to validate the feasibility and performance of the MWCE. Therefore, ex-vivo trials will be also validated on a fresh porcine stomach from Sheng Siong Supermarket Pte Ltd (7 Jurong West Avenue 5, Singapore 649486, geographic coordinate 1°20'55.7"N 103°42'13.5"E) [33]. The proposed MWCE is also filled with 1.084ml of CA (60%) and 0.4326 PB in the acid chamber and the base chamber, respectively. It can be observed that the proposed MWCE could open the inflation valve and deflation valve as desired. The outer balloon was successfully inflated and deflated not only in a virtual stomach environment but also in a real porcine stomach. The images for the inflation stage of the balloon given in the Figs 11 and 12 were taken approximately 15 minutes after the inflation valve was opened. The inflated balloon volume was measured using the experimental setup given in previous section. The inflated balloon volumes from the virtual stomach experiment and the porcine experiments are approximate 171ml (*D*_*balloon*_ = 68.86mm) and 169.8ml (*D*_*balloon*_ = 68.7mm), respectively. It can be seen that the balloon diameter is always greater than 40mm. Hence, the balloon is guaranteed to stay inside the human stomach without passing through the pyloric sphincter.

## Discussion

IGBs have been shown to be effective at inducing weight-loss greater than life style therapy or pharmacotherapy. Compared to surgical treatment, IGB can be attractive to patients as it is less invasive than surgery. To maximize the safety and to prevent unduly long exposure of non-responding patients to a potentially hazardous treatment, the balloon should be removed every 3 months and strict weight-loss is defined as a continuation of treatment [34]. However, it is generally recommended that IGB can be also removed within 6 months [35]. Only the Spatz Adjustable Balloon System is allowed for a 12-month period [36]. The treatment period ranges from 3 months to 1 year depending on the patients’ condition. Carbonelli et al. [37] described that the majority of patients have successfully achieved weight loss and continue to lose weight after the removal of the IGB. Mathus-Vliegen et al. [34] noted that 55% of patients maintained or continued losing 10% weight after 1 year-post removal. According to a survey reported by Dumonceau et al. [6] among 4371 patients of 22 non-randomized studies, the mean weight loss was 17.8 kg for the first year treatment. However, 20%-40% of patients fail to lose weight due to the early removal of IGB. Solmaz et al. [38] reported a lost mean of 7.38 kg at the end of first month and 14.4 kg at the end of 6 months for obese patients using the IGB. The loss of IGB efficacy after 3 months has been demonstrated convincingly in many studies and it is considered as a major drawback [39]. In recent study, it has been recommended that a placement of second balloon 1 month after the removal of the first balloon should be carried out. For example, Lopez-Nava et al. [40] indicated an additional drop of 2.6 points of BMI in 112 patients following consecutive balloon placements. Genco et al. [41] reported a further drop of 3.9 points of BMI after the second balloon placement. Dogon [42] reported that successful weight loss of 96, 71, and 50% was obtained in at 6, 12, and 18 month, respectively. For patients losing ≥ 5% of their initial weight after 1 month of balloon treatment, they recognized that the treatment can achieve a significant weight loss and maintain it 1 year after the IGB removal. For our proposed capsule balloon, it is recommend that the treatment period should be ranged from 6 months to one year and the balloon should be removed every three months and replaced with a new one.

Using the proposed MWCE, it has been shown that an outer balloon can be successfully inflated to a desired volume and deflated to its original shape after a given period of time. The proposed magnetic capsule demonstrates a number of advantages over existing weight-loss capsule robots. First, a spread of experiments across the different acid-base combinations to determine the smallest volume of aqueous acid and solid base has been introduced. As a result, a guideline for the optimal volume ratio of the chemical chambers can be obtained. Compared with other similarly sized weight-loss capsules, our proposed MWCE produces a larger volume of the CO_2_ gas as it has more space for the chemicals due to the reduced size of the activation mechanism. Second, compared withother approaches of the magnetic capsule robots in the literature [15–17], our proposed MWCE does not require any precise positions/orientations and force for the operation. Hence, it provides simpler, safer, and easier ways for the practical implementation of both ex-vivo and in-vivo experiments. In addition, the smaller size of the capsule means easier and more comfortable ingestion for the patients. From the experimental results and observations during the development of the proposed MWCE, a few issues worth noting in clinical implementations are still opened for discussion and further development.

In our current approach, the proposed MWCE is assumed to reach the patient stomach with no prior position information. It has been reported in the literature that a simple sensor network can be easily designed to estimate the magnetic capsule position [43–45]. For safety, the MWCE position should be determined using these sensor networks. The magnetic fields from the capsule’s internal magnets allow the external sensor network to detect its position once it has reached the patient’s stomach. A few millimetres of error are acceptable. Radiofrequency identification (RF) or X-rays may also be used to confirm the position of the device as the magnets are strongly radiopaque. The position information of the MWCE can be directly linked to smart electronic devices or even smartphones. This allows convenient and comfortable use for the patient. In order to determine the inflation state of the outer balloon, the swallowed magnetic capsule should be monitored from 6 to 8 hours using the magnetic sensor network to determine its position in the stomach. If the balloon is inflated, its outer balloon size is bigger than the pyloric sphincter and it is guaranteed to stay inside the patient’s stomach. In addition, a sufficient amount of the methylene blue powder could be placed inside the balloon. If the balloon is suddenly broken, the blue powder will be released into the stomach environment and the patient’s urine colour will be changed. For deflation state, the magnetic sensor network can also be used to confirm the capsule position. Once the balloon is deflated, it will be compacted to a small size and easily passes through the pyloric sphincter.

During the validation, a latex balloon was used to represent the IGB. In real clinical applications, biocompatible balloons have to be used. Currently, the materials of the commercial IGBs are made by silicone, thermal plastic polymer or polyurenthane. One of the main challenges for current IGBs is the large thickness of the balloon wall to contain high-inertia fluids like water. To optimise the space in our capsule, a further reduction in the balloon thickness is necessary. However, a reduction of the balloon thickness can increase its permeability to CO_2_ gas and causing the balloon to deflate after few days. One of the possible methods to overcome diffusion losses is to combine two thin layers for the designed balloon. An inner thin layer of non-stretch reduced permeability packaging film (eg. EVAL by Kuraray) is inserted into an outer thin biocompatible elastic balloon to form a new IGB. The outer elastic balloon will compress the inner layer, which is pre-pleatedinto a compact wrapper around the capsule. Theoretically, the new balloon structure is able to reduce the leakage of CO_2_ gas. In practice, more experimental validations are needed for the new design of the IGB. The current maximum diameter of the proposed MWCE is normally less than the minimum width of the oesophagus. In order to further improve the patient’s convenience, the MWCE and its outer balloon can be coated by a biodegradable material like chitosan or gelatine. Coatings to facilitate the passage of the capsule and maintain its integrity could be considered. For example, a gelatine coating would keep the membrane from flapping as it passes down the oesophagus.

The measured volumes of the outer balloon using the proposed MWCE are smaller than that in real time measurement of the chemical reaction experiments. The main reason is due to the sudden increase in the pressure and the gas generated from the reaction. Some acid is forcefully ejected through small holes in the base chamber, leaving behind unreacted base in the capsule To overcome this problem, around 10% to 15% of the base amount should be put outside the capsule. Once the acid is ejected from the inner chambers of the capsule, it will react with the external base and therefore the reaction will be more complete. In addition, the exhaust holes could be made bigger and few holes could be created in the cover head of the base chamber. These modifications will improve the reaction performances and more CO_2_ will be produced. As observed from the trials 4 and 5 given in Table 3, when we placed around 10% of the base powder outside the capsule chamber and made bigger exhaust holes as well as added holes in the cover head of the base chamber, the measured volumes of the outer balloon are bigger compared to the first three trials. The ex-vivo experiments were also carried out with the modified capsule and the results showed bigger volumes for the inflated balloon (171ml for virtual stomach experiment and 169.8ml for porcine stomach experiment).

The clinical considerations that could impact the oral administration of the capsule have to do with the shape of the stomach, the rate of gastric emptying, and the minimum distance from the stomach to the abdominal wall. More studies on the stomach anatomy of obese patients in terms of measurements of distance from the stomach to the abdominal wall and their stomach shape should be carried out in order to determine the external magnet size. The shape of the stomach could have an effect on the reliability of the actuation mechanism. To avoid the capsule getting trapped in folds of the stomach lining, the patient should ingest around 300ml to 500ml of water along with the capsule so as to partially expand the stomach. This allows the capsule to freely rotate and anchor itself onto the stomach wall in the right orientation when the external magnet is brought close to the patient’s stomach. Hence, the capsule needs to be inflated while the stomach is still in a distended state. In normal patients, large food particles arriving in the stomach start being evacuated through the pyloric sphincter after a lag time of about 15–20 minutes [46], whereas water starts draining away almost immediately [47]. Thus, the capsule should achieve sufficient size by the 15 minutes to prevent it from passing through the pyloric sphincter. However, the rate of gastric emptying varies among the population. It is also known from clinical experiences that the presence of food or other mushy substances will obstruct the exit of the capsule [48]. However, solid detritus may adversely affect the free locomotion of the capsule which is necessary for the inflation mechanism. It was thus ruled out as a solution. The position of the patient was looked at as a possible modulator of the residence time. It is known that making the patient lie on his/her right side (right lateral recumbent position) increases the rate of gastric emptying [49]. Thus, a standing or left lateral recumbent position could be adopted instead. The buoyancy of the capsule also determines which position is best. If the capsule is denser than the stomach fluids, then an erect position should be avoided since it will sink closer to the pylorus.

## Conclusion

This paper introduces a new approach for the obesity treatment using a new magnetic weight-loss capsule robot. The magnetic capsule is able to inflate and deflate a latex balloon to a desired volume. It offers a safer and less invasive method for losing weight as compared to other weight-loss methods and existing weight-loss capsule robots in the literature. Oral administration eliminates hospitalization and recovery time, eliminates complications related to endoscopy or surgery, and mitigates long-term side effects from drugs. In addition, our design magnetic weight-loss capsule provides more effective weight-loss due to its dynamic administration process. In summary, our proposed solution significantly improves patient experience and safety comparing to other available solutions. The design is also simple and low cost, making it a very viable commercial product. Future research activities will concentrate on the validation on the living animal and human. The balloon materials and relevant optimizations will be also carried out in order to fulfil the art for the magnetic weight-loss capsule robot.
