# Correction: Development and Testing of a Magnetically Actuated Capsule Endoscopy for Obesity Treatment

**DOI:** 10.1371/journal.pone.0151711

**Published:** 2016-03-10

**Authors:** Thanh Nho Do, Tian En Timothy Seah, Khek Yu Ho, Soo Jay Phee

The third author’s name is spelled incorrectly. The correct name is: Khek Yu Ho. The correct citation is:

Do TN, Seah TET, Ho KY, Phee SJ (2016) Development and Testing of a Magnetically Actuated Capsule Endoscopy for Obesity Treatment. PLoS ONE 11(1): e0148035. doi:10.1371/journal.pone.0148035
